# The effects of short-term light exposure on subjective affect and comfort are dependent on the lighting time of day

**DOI:** 10.1038/s41598-021-81182-y

**Published:** 2021-01-28

**Authors:** Lijun Chen, Fang-Fang Yan, Shuhan Fan, Yifan Wu, Jia Yang, Hua Yang, Chang-Bing Huang

**Affiliations:** 1 CAS Key Laboratory of Behavioral Science, Institute of Psychology, Chinese Academy of Sciences, Beijing, 100101 China; 2 Department of Psychology, University of Chinese Academy of Sciences, Beijing, 100049 China; 3 Institute of Semiconductors, Chinese Academy of Sciences, Beijing, 100083 China

**Keywords:** Psychology and behaviour, Psychology, Environmental impact

## Abstract

Light, one of the key environmental components for both life and work, played significant role in subjective feelings (e.g. affect and comfort), but the exact effects and mechanisms were still to be determined. The present study screened thirty healthy adults (13 females, 22.45 ± 3.26 years) and examined subjective affect and comfort under short-term white lights with different combination of correlated color temperature (CCT) and illuminance at different times of day (e.g. morning, afternoon, and evening). Our results showed a significant interaction between illuminance level and time-of-day on subjective comfort. Participants felt more comfortable under 50 lx and 100 lx instead of 500 lx in the evening, and more comfortable under 500 lx in the morning and afternoon. In addition, a positive correlation between illuminance and comfort in the morning and a negative correlation between them in the evening were found. No significant effect of CCT on any subjective feeling was revealed. Our results necessitate the consideration of time-of-day in understanding lighting effects and application of healthy lighting in daily life.

## Introduction

Apart from significant role in inducing visual effects, light also plays a critical role in non-visual functions, e.g. subjective affect and comfort^1^. Since Kripke et al. (1983) found that exposure with high-intensity light can reduce the depression symptoms in patients with depressive disorder^2^, light had been widely used to treat affective disorder in clinical practice^3–5^. In recent years, researches have also suggested significant effects of light on subjective affect and comfort in normal population^1,6–8^, which provided possibilities for using light to improve subjective well-being in daily life and attracted sustainable attention in the past decades.

Illuminance and spectral distribution are two commonly studied properties of light in modulating subjective affect and comfort, dated back to the influential study by Kruithof who presented a graph to show preferred interior lighting conditions in terms of illuminance and correlated color temperature^9^. According to the Kruithof’s rule, people felt more comfortable and pleasing at low illuminance and low CCT light condition and at high illuminance and high CCT condition. The Kruithof’s rule was widely applied in interior lighting design, although subsequent studies were highly controversial^10–14^. For example, Park et al. (2013) found a significant effect of illuminance but not CCT on pleasant feeling, at which subjects felt more pleasant at high (600 lx) instead of low illuminance (300 lx) condition^13^. On the contrary, Sunwoo et al. (2017) found similar effect of high and low illuminance (700 lx vs. 300 lx) on affect under short-term light exposure (~ several minutes). In the study of the relationship between spatial distribution of light and subjective affect and comfort, lower CCT was endorsed by some researches e.g.^1,15–17^ while higher CCT was favored by others' e.g. ^7,18–20^. For example, Sunwoo et al. (2017) found a significantly positive effect of lower CCT (3000 K) on subjective feelings (e.g. felt more comfort and happiness), as opposed to 6500 K, but Chellappa et al. (2011) found that exposure to a higher CCT condition (6500 K) for 2 hours before bedtime strongly suppressed the secretion of melatonin and enhanced subjective well-being (e.g. mood, tension and physical comfort), as opposed to the 2500 K CCT condition. In addition, many studies failed to find the effect of CCT on subjective comfort and affect^13,21^.

Factors such as lighting duration and timing may contribute to the conflicting results. At one hand, lighting duration ranged from several minutes^1^ to hours^22^ in previous laboratory studies and could last even several weeks in field studies^18^. On the other hand, subjects can be exposed to light in the morning (e.g. 08:00;^23^), afternoon (e.g. 15:00;^13^), and/or evening (e.g. before sleep;^19^). Anecdotal evidence has suggested different timing of lighting may have reverse effect, e.g. advancing or delaying circadian phase^24^. Also, the majority of researches about the effects of light were carried out together with cognitive tasks, e.g. go/no-go, *N*-back working memory, Psychomotor Vigilance Task, Flanker task, long-term memory, and emotional judgment task^13,16,17,22,25^. The tasks performed during light exposure and associated (potential) fatigue might also confound the effects of light on subjective affect and comfort^26^.

Previous studies had confirmed the effectiveness of short-term light exposure on modulating physical and psychological processes^8,13,27,28^. In the current study, we examined the effects of short-term light exposure with different combination of illuminance, CCT, and exposed time of day on subjective affect and comfort to minimize the confounding influence of fatigue on subjective feelings. We also spared subjects during light exposure, aiming to isolate “pure” effects of light on subjective affect and comfort. We found significant interaction between the illuminance level, but not CCT, and time-of-day on subjective comfort. Our results suggested that research of lighting effects and design of healthy lighting should take into account the influence of lighting time of the day.

## Results

The results of multivariate analysis of variance (MANOVA) revealed significant main effect of time-of-day on arousal (F(2,241) = 5.572, *p* = 0.004, Partial η^2^ = 0.044) and interaction of illuminance and time-of-day on comfort (F(4,241) = 3.980, *p* = 0.004, Partial η^2^ = 0.062). All other interactions were not significant (Table 1).

**Table 1:** MANOVA analysis of CCT, illuminance, and time-of-day on affect and comfort.

	df	MS	F	*p*	Partial η^2^
**CCT**					
Valence	2	2.291	1.670	0.190	0.014
Arousal	2	0.195	0.115	0.892	0.001
Dominance	2	0.906	0.411	0.664	0.003
Comfort	2	0.401	0.729	0.483	0.006
**Illuminance**					
Valence	2	0.775	0.565	0.569	0.005
Arousal	2	2.242	1.318	0.269	0.011
Dominance	2	1.140	0.517	0.597	0.004
Comfort	2	0.553	1.006	0.367	0.008
**Time-of-day**					
Valence	2	0.557	0.406	0.667	0.003
Arousal	2	9.478	5.572	0.004	0.044
Dominance	2	6.540	2.966	0.053	0.024
Comfort	2	1.463	2.663	0.072	0.022
CCT * Illuminance					
Valence	4	0.193	0.141	0.967	0.002
Arousal	4	0.316	0.186	0.946	0.003
Dominance	4	0.338	0.153	0.961	0.003
Comfort	4	0.130	0.237	0.917	0.004
CCT * Time-of-day					
Valence	4	0.034	0.025	0.999	0.000
Arousal	4	0.955	0.561	0.691	0.009
Dominance	4	0.177	0.080	0.988	0.001
Comfort	4	0.147	0.268	0.898	0.004
Illuminance * Time-of-day					
Valence	4	0.499	0.364	0.834	0.006
Arousal	4	0.133	0.078	0.989	0.001
Dominance	4	0.625	0.283	0.889	0.005
Comfort	4	2.186	3.980	0.004	0.062
CCT * Illuminance * Time-of-day					
Valence	8	0.442	0.323	0.957	0.011
Arousal	8	0.594	0.349	0.946	0.011
Dominance	8	0.187	0.085	1.000	0.003
Comfort	8	0.241	0.438	0.897	0.014

MS: mean square; F, *p*, and partial η^2^ refer to the F value, significance level, and effect size from the MANOVA analysis, respectively.

**Figure 1:**
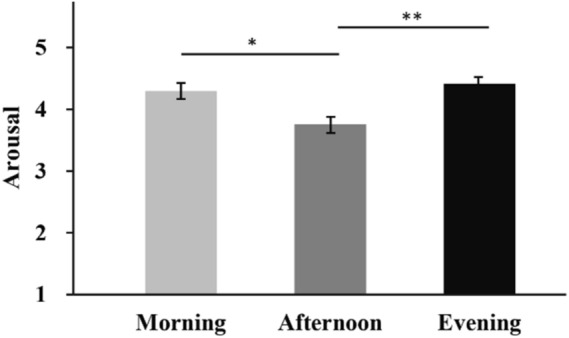
Pairwise comparisons for arousal level among different time-of-day. *p < 0.05; **p < 0.01.

For the effect of time-of-day on arousal level, post-hoc tests found that participants felt more arousal in the morning (4.296 ± 0.130, mean ± s.e.; 95% Confidence Interval (CI) = [4.037, 4.555]; *p* = 0.011) and evening (4.416 ± 0.132, 95% CI = [4.152, 4.679]; *p* = 0.002) than in the afternoon (3.756 ± 0.132, 95% CI = [3.496, 4.017]; Fig. 1).

**Figure 2:**
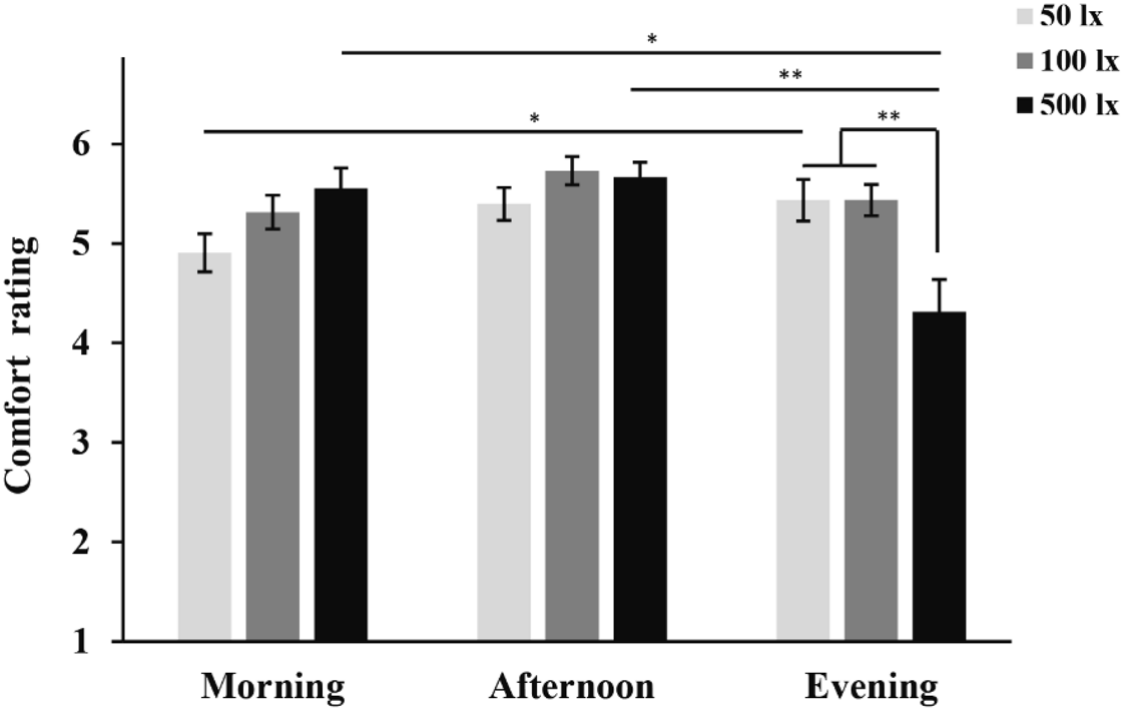
Pairwise comparisons among comfort ratings under different illuminance levels and time-of-day. *p < 0.05; **p < 0.01.

The simple effect of illuminance on comfort was found in the evening session (F(2,241) = 6.890, *p* = 0.001, Partial η^2^ = 0.054; Table 2). Post-hoc tests found that participants felt more comfortable under 50 lx (5.435 ± 0.209, 95% CI = [5.001, 5.869]; *p* = 0.003) and 100 lx (5.5438 ± 0.157, 95% CI = [5.112, 5.763]; *p* = 0.007) than 500 lx (4.313 ± 0.327, 95% CI = [3.637, 4.988]; Fig. 2). The simple effects of time-of-day on comfort were found at 50 lx (F(2,241) = 3.602, *p* = 0.029, Partial η^2^ = 0.029) and 500 lx (F(2,241) = 5.960, *p *= 0.003, Partial η^2^ = 0.047; Table 2). Post-hoc tests showed that subjects felt more comfortable in the evening (5.435 ± 0.209, 95% CI = [5.001, 5.869]), as opposed to morning (4.907 ± 0.191, 95% CI = [4.516, 5.299]; *p* = 0.031) under 50 lx and more comfortable in the morning (5.556 ± 0.202, 95% CI = [5.140, 5.971]; *p* = 0.042) and afternoon (5.667 ± 0.150, 95% CI = [5.364, 5.970]; *p* = 0.002), as opposed to evening (4.313 ± 0.327, 95% CI = [3.637,4.988]) under 500 lx (Fig. 2).

**Table 2:** The simple effect of illuminance and time-of-day on comfort.

	df	MS	F	*p*	Partial η^2^
**Time-of-day**					
Morning	2	0.785	1.430	0.241	0.012
Afternoon	2	0.137	0.250	0.779	0.002
Evening	2	3.785	6.890	0.001	0.054
**Illuminance**					
50 lx	2	1.979	3.602	0.029	0.029
100 lx	2	0.532	0.968	0.381	0.008
500 lx	2	3.274	5.960	0.003	0.047

MS: mean square; F, *p*, and partial η^2^ refer to the F value, significance level, and effect size from the MANOVA analysis, respectively.

**Figure 3:**
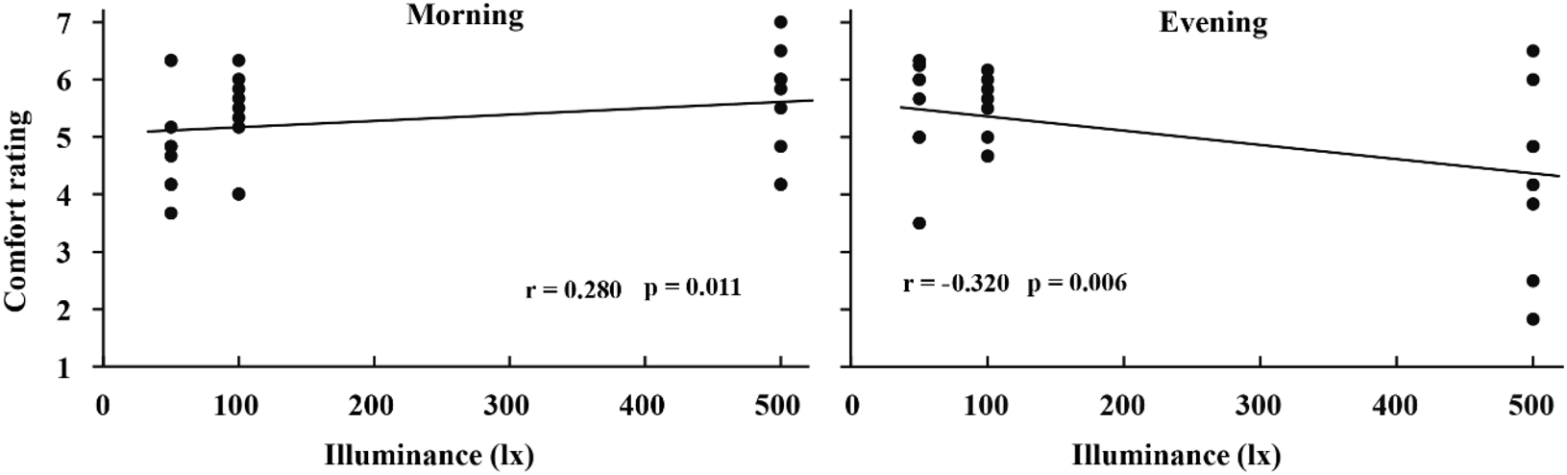
Correlation between illuminance and comfort in the morning (left) and the evening (right).

To further explore the relationship between illuminance and comfort, a Spearman correlation analysis was performed. Interestingly, a positive correlation (r = 0.280, *p* = 0.011) was found between illuminance and comfort in the morning while a significantly negative correlation (r = − 0.320, *p* = 0.006) between them in the evening (Fig. 3).

## Discussion

A significant main effect of time-of-day on arousal level was found in the current study. We didn’t find any significant effect of CCT and illuminance on the affective states (e.g. valence, arousal, dominance) under short-term light exposure (e.g. 3 min). Interestingly, a significant interaction between illuminance and time-of-day on subjective comfort was detected. Further analysis revealed a dissociated relationship between illuminance and comfort in the morning and evening.

Previous studies have also revealed significant effect of time-of-day on subject’s arousal level^29,30^. For example, the work performed by Parrott who assessed the arousal in different groups (e.g. sedative smokers, stimulant smokers, nicotine deprived smokers and non-smokers) at different times of day showed that feelings of arousal were significantly affected by time. The result that all groups started the day after waking with low arousal but peaked at different times suggested that even though the changing patterns over time differed between subgroups, the arousal was significantly affected by time of day^29^. Our result that participants felt more arousal in the morning and evening also suggested this point.

In the present study, the effect of illuminance, instead of CCT, on subjective comfort was found to be significant. Our results were inconsistent with the Kruithof’s rule that suggested the preferred interior lighting conditions with combinations of illuminance and CCT^9^ while partially consistent with some other researches that only found significant effect of illuminance on subjective comfort. According to Boyce’s work, people felt more pleasant and comfortable in light condition with higher illuminance level while the CCT didn’t show significant effect^10^. Fotios revised the Kruithof’s curve and suggested that the variation in CCT has a negligible effect on subjective pleasure and the one condition to avoid is low illuminance less than 300 lx^12^. On the other hand, our results indicated the effects of illuminance on subjective comfort was affected by the lighting time of the day: subjects felt more comfortable in higher illuminance level in the morning, and low illuminance in the evening.

Light is one of the major cues to discriminate day and night and keep normal circadian rhythm^31,32^. Exposure to less light at daytime and bright light in the evening can impact circadian rhythm negatively^33,34^. We argue that light echoed with natural circadian may inherently link to more positive effects^13^ and our observation of the significant interaction of illumination and time of day on affect and subjective comfort necessitated the consideration of temporal aspects of light exposure in the understanding of lighting effects and the design of healthy lighting.

To minimize the potential confounding of fatigue on subjective feelings, the present study adopted a short-term light exposure. However, compared to short-term exposure, long-term exposure to light might have a different or even a reverse effect on cerebral activity. For example, a research conducted in 2017 found that short-term exposure to monochromatic blue light decreased lower-frequency electroencephalogram bands while long-term exposure induced an increase in lower frequency bands^35^. The effects of long-term light on subjective affect and comfort should be investigated in future.

## Conclusions

In summary, the present study reported a significant interaction between light illuminance and time-of-day on subjective comfort. Our results contribute to the understanding of light effect and will be of interest to the design of healthy lighting environment in daily work and life.

## Methods

### Participants

Thirty healthy adults, aged 18 to 31 years (13 females, 22.45 ± 3.26 years), participated in the study and received monetary compensation. All subjects had normal or corrected-to-normal visual acuity, normal color vision, and were free of ocular diseases. All participants gave their written informed consents before experiment began. The study was approved by the local ethical committee of Institute of Psychology, Chinese Academy of Sciences and all research activities were adhered to the principles of the Declaration of Helsinki.

### Study design

**Figure 4:**
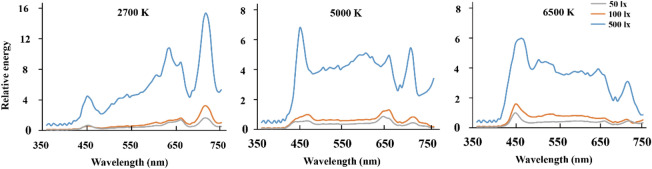
Relative spectral distribution of different light conditions: left: 2700 K, middle: 5000 K; right: 6500 K; Gray: 50 lx; Orange: 100 lx; Blue: 500 lx.

The experiment employed a 3 × 3 × 3 mixed-group design with three independent variables: illuminance (50 lx vs. 100 lx vs. 500 lx, at 0.75 m height table level) and CCT (2,700 K vs. 5,000 K vs. 6,500 K) as within-subject factors, and time-of-day (morning vs. afternoon vs. evening) as between-subjects factor. The relative spectral distribution of the nine light conditions was shown in Fig. 4. The morning session started at 9:30 a.m., the afternoon session started at 3:00 p.m., and the evening session started at 7:30 p.m. Constrained by the availableness of subjects’ schedule, nine, thirteen, and eight subjects were assigned to the morning, afternoon and evening sessions, respectively. Each participant went through all nine light conditions and every condition was repeated twice. The testing order of all light conditions was counterbalanced across subjects.

**Figure 5:**
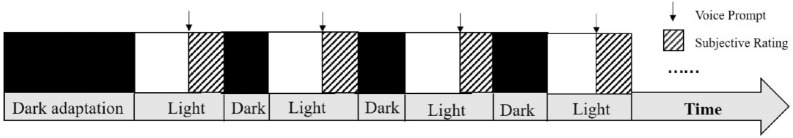
Testing sequence. Subjects were exposed to different lights one after another with a 2-min dark interval (< 0.01 lx). After light exposure, a brief voice (indicated by arrow) prompted participants to rate their current subjective feelings within 1 min under the same light condition.

For each subject, after 5-min dark adaptation (< 0.01 lx; Fig. 5), different lights were presented one after another with a 2-min dark (< 0.01 lx) interval in-between. In each trial, participants were exposed to one light condition for 3 min, during which subjects were asked to sit still in the first 2 min and rate their current subjective feelings after a voice prompt in the last 1 min. Subjects were informed to sit quietly without closing eyes during exposure.

### Environmental setting

The study was carried out in a windowless room, with a size of 4.36 × 4.03 × 2.70 m. The walls and ceiling of the room were white. Six identical custom-made LED lighting devices, with spectrum ranged from 380 to 780 nm and surface homogenized by scatter plate, were mounted onto the ceiling. Each LED lighting device consisted of 22 classes of monochrome LEDs and two classes of white LEDs. The intensity of each class of the LED can be adjusted for different combinations of spectral intensity with control accuracy better than 1%. The device can simulate most natural lighting conditions, in which the desktop illuminance of typical CIE white illuminant can reach 750 lx and correlated color temperature can cover from 1800 to 7500 K.

### Assessment of subjective feelings

Subjective affect states, comfort, and eye fatigue were evaluated under each light condition. Participants rated their affects in terms of valence, arousal, and dominance with 9-point Self-Assessment Manikin Test^36^, with 1 for unpleasant, calm, and controlled state, and 9 for pleasant, excited, and in-control state. The subjective comfort and eye fatigue under each light condition were rated via a 7-point scale, with 1 for discomfort or non-fatigue to 7 for comfort or eye fatigue.

### Statistical analysis

All analyses were performed by SPSS version 20 (IBM, USA). The rating scores of subjective feeling for each light condition were averaged for each subject. The effects of time of day (morning vs. afternoon vs. evening; between-subject factor), CCT (2700 K vs. 5000 K vs. 6500 K; within-subject factor), and illuminance (50 lx vs. 100 lx vs. 500 lx; within-subject factor), and interactions of the three factors on subjective feelings, e.g. valence, arousal, dominance, and comfort, were computed using multivariate analysis of variance (MANOVA), with eye fatigue as covariate. Post-hoc tests of simple effects were performed with Bonferroni correction^37^. Non-parametric correlation analysis was performed to test the relationship between illuminance and comfort in different times of day.

## Data Availability

The data that support the findings of this study are available from the corresponding author upon reasonable request.
